# Phenotypic Stability of Sex and Expression of Sex Identification Markers in the Adult Yesso Scallop *Mizuhopecten yessoensis* throughout the Reproductive Cycle

**DOI:** 10.3390/ani9050277

**Published:** 2019-05-24

**Authors:** Kazue Nagasawa, Tongchai Thitiphuree, Makoto Osada

**Affiliations:** Laboratory of Aquacultural Biology, Graduate School of Agricultural Science, Tohoku University, 468-1 Aoba, Aramaki, Aoba-ku, Sendai, Miyagi 980-8572, Japan; nungava@hotmail.com

**Keywords:** bivalve, mollusk, reproduction, gonochorism, protandry, *Patinopecten*

## Abstract

**Simple Summary:**

Bivalve sex is thought to fluctuate depending on environmental conditions. So far, there has been no investigation on the phenotypic stability of sex in the commercially important Yesso scallop *Mizuhopecten yessoensis*. The present study revealed that the sex of the Yesso scallop is stable after initial sex differentiation and that this species maintains a sex-stable maturation system throughout its life. In addition, gonad differentiation for each sex was precisely characterized by using molecular markers throughout the maturational cycle.

**Abstract:**

The objective of the present study was to analyze the phenotypic stability of sex after sex differentiation in the Yesso scallop, which is a gonochoristic species that has been described as protandrous. So far, no study has investigated in detail the sexual fate of the scallop after completion of sex differentiation, although bivalve species often show annual sex change. In the present study, we performed a tracking experiment to analyze the phenotypic stability of sex in scallops between one and two years of age. We also conducted molecular marker analyses to describe sex differentiation and gonad development. The results of the tracking experiment revealed that all scallops maintained their initial sex phenotype, as identified in the last reproductive period. Using molecular analyses, we characterized *my-dmrt2* and *my-foxl2* as sex identification markers for the testis and ovary, respectively. We conclude by proposing that the Yesso scallop is a sex-stable bivalve after its initial sex differentiation and that it maintains a sex-stable maturation system throughout its life. The sex-specific molecular markers identified in this study are useful tools to assess the reproductive status of the Yesso scallop.

## 1. Introduction

Systems of sex differentiation and phenotypic stability of sex have evolved into diverse forms in molluskan species and often show species-specific features. Owing to the diversity of sex-controlling systems, there is still controversy about the molecular mechanisms of sex differentiation, particularly in bivalves. The Yesso scallop (*Mizuhopecten yessoensis*, previously called *Patinopecten yessoensis*) is a gonochoristic species that has been described as protandrous [1]. Previous studies [2,3,4,5] have proposed that all juveniles first differentiate into males possessing a small amount of sperm, and then, in the next reproductive season, some of the males undergo a sex change to female via a hermaphroditic transition phase. The sex differentiation of the Yesso scallop is generally completed within one year in most parts of Japan where aquaculture of this species is performed [5]. However, this hypothetical protandrous model may require further validation because Maru [6] pointed out that no study has confirmed whether the hermaphroditic gonad eventually transforms into the ovary of a female. Interestingly, many population-based studies [2,4,5] have reported that after the completion of sex reversal, the Yesso scallop (1–5 years of age) normally showed a sex ratio of approximately 1:1 in various culture conditions, while hermaphroditic gonads were very rarely observed [3]. These findings imply that sex determination in the Yesso scallop is strictly controlled by genetic factors rather than by environmental ones. These observations suggest that the Yesso scallop has a system that firmly regulates phenotypic sex consistently throughout life, even though no sex chromosomes have yet been identified in mollusks.

To understand the phenotypic stability of sex in bivalves, Park et al. [7] directly confirmed annual sex reversal in the Pacific oyster (*Crassostrea gigas*) by tag tracking. They confirmed that sub-populations of both males and females can re-change sex after the first sex reversal. In fact, the results revealed that the phenotypic sex of the Pacific oyster fluctuates annually and is regulated by external factors (i.e., temperature, food availability, exogenous steroids, and pollutants), as reviewed elsewhere [8]. Thus, it can be said that the Pacific oyster has high plasticity of sex differentiation throughout its life. In addition, a similar annual sex change has been reported in a bloody clam (*Tegillarca granosa*) [9]. For scallops, Coe [10] reported variations in sexuality of several European species of *Pecten* that are mostly hermaphroditic. In addition, a recent book [1] reviewed sexuality in various scallops that show species-specific varieties in sexuality. However, to the best of our knowledge, no study has investigated the phenotypic stability of sex in the Yesso scallop, despite the strict control of sex determination mentioned above. Therefore, we aimed to analyze the phenotypic change of sex after the first sex reversal in the Yesso scallop by performing a tag-tracking experiment. In previous studies, the judgment of sex was performed by histological observation. After sex differentiation, the seasonal changes of gonad development were classified into seven stages [4,11,12]. During the reproductive period, the sex of an adult scallop can be determined by visual judgment of gonad color. Therefore, the Yesso scallop is a good model species that can be used for understanding the mechanism of bivalve reproduction [11,13]. During the annual sex-differentiating phase, however, both males and females exhibit immature and transparent gonads, wherein a very small amount of undifferentiated germ cells exists (i.e., spermatogonia for males or oogonia for females), which are not distinguishable histologically [8]. Hence, thus far, it has not been possible to perform definitive judgments of sex during the sex-differentiating period at every age. Therefore, a sex-specific molecular marker is required to confirm the sex in order to research sex differentiation during gonad development in the Yesso scallop.

In vertebrates, the early stages of gonad sex differentiation have been described using several molecular markers, alongside studies in teleosts [14]. Ijiri et al. [15] confirmed the identity of sex differentiation-related molecules (i.e., steroidogenic enzymes, steroid receptors, transcription factors, and anti-Mullerian hormone (Amh) during gonadal differentiation and development) in tilapia, which is a gonochoristic fish with an XX/XY sex-determining system. Doublesex/male-abnormal-3-related transcription factor 1 (Dmrt1) exhibited male-specific expression, suggesting that it plays an important role in testicular differentiation. Indeed, Dmrt-related molecules have been found in several bivalves and are thought to be key players in male gonadal development (as in the case of vertebrates) in Pacific oyster [16], *Chlamys nobilis* [17], lion-paw scallop (*Nodipecten subnodosus*) [18], and Akoya pearl oyster (*Pinctada fucata*) [19]. In ovarian differentiation, Nagahama [20] found that the forkhead transcription factor (Foxl2) activates *P450arom* transcription to lead to ovary differentiation, whereas Dmrt1 inhibits this in teleosts. In addition, sexually dimorphic expression of *foxl2* has been reported in bivalves, namely, *Chlamys farreri* [21] and pearl oyster (*Pinctada margaritifera*) [22]. In the Yesso scallop, Li et al. [23] recently developed a method for scallop sex identification during the sex-differentiating phase by using LOG_10_(*DMRT1L*/*FOXL2*). Therefore, mechanistically, the involvement of Dmrt and Foxl2 could be critical for testis and ovary differentiation, respectively, after the completion of sex differentiation in the Yesso scallop. There is no information regarding the phenotypic stability of sex in the Yesso scallop after sex differentiation, even though this scallop is a good model species for the analysis of phenotypic sex.

## 2. Materials and Methods

### 2.1. Tracking Experiment for the Analysis of Phenotypic Stability of Sex in the Yesso Scallop

**Figure 1:**
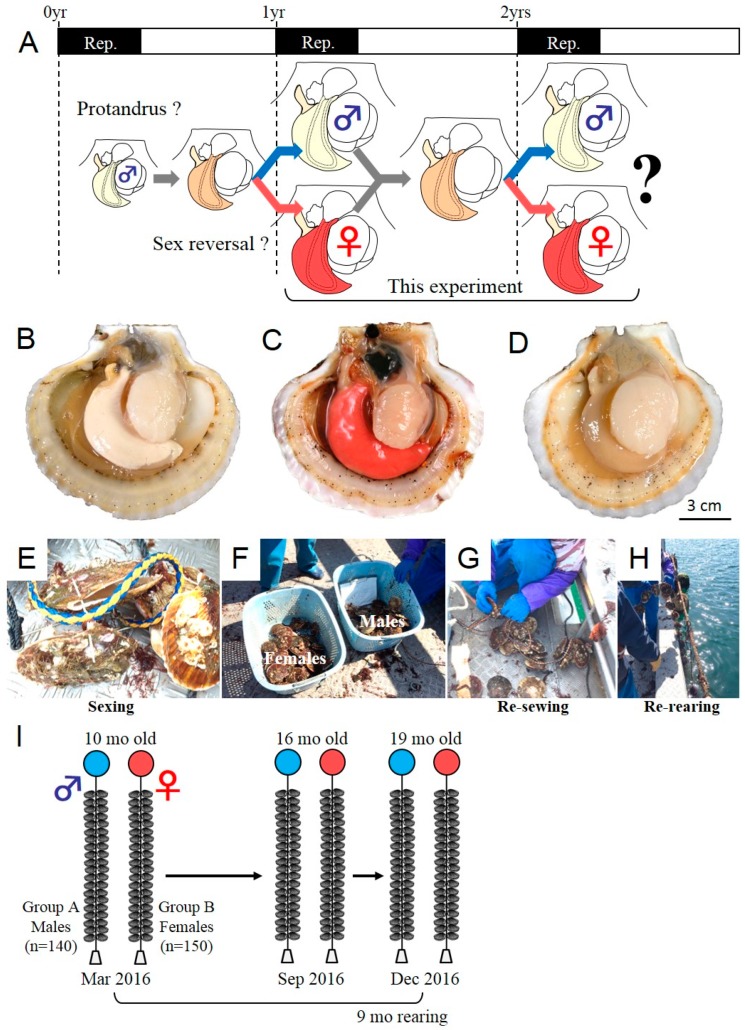
Overview of the analysis of phenotypic stability of sex in the adult Yesso scallop. (**A**) Diagrammatic illustration of sex differentiation in the Yesso scallop. The present study focused on the stage of sex differentiation from 1 to 2 years of age. (**B–D**) Representative dissection images of adult scallops at two years of age. In the reproductive (Rep) phase, males exhibit a milky-colored gonad (**B**), whereas females have an orange/red-colored gonad (**C**). In the non-reproductive phase, the sex is not distinguishable by observation of gonad color (**D**). Hence, sexing was performed during the reproductive period. (**E–I**) Tracking experiment for the analysis of phenotypic stability of sex. (**E**) Sexing was performed by checking gonad color. (**F**) Males and females were divided into two groups. (**G–I**) Each sex group was reattached to different rearing ropes (**G**) and reared again for extra nine months until the next reproductive period (**H,I**).

In March 2016, 10-month-old farmed Yesso scallops (*M. yessoensis*) were purchased (approximately 300 scallops) from a local commercial supplier (Ogatsu Bay, Miyagi, Japan). This population in Miyagi Prefecture already undergoes sex differentiation to female or male at 10 months of age (Figure 1A). This scallop species has a clear reproductive period in spring, as described elsewhere [11]. During this reproductive period, the gonads exhibit a sex-specific color (i.e., milky white color for testis in males, as shown in Figure 1B, orange-red color for ovary in females, as shown in Figure 1C). Meanwhile, in summer, male and female gonads exhibit the same color (i.e., beige color for both sexes) during the spent stage (Figure 1D) and are not distinguishable histologically. For the tracking experiment, sexing was performed for all individuals in March 2016 (10 months of age) by visual judgment of gonad color on shore (Figure 1E). If there was uncertainty about the sex, we discarded the scallops (*n* < 5). In total, male (*n* = 140) and female scallops (*n* = 150) were identified, divided into two groups (Figure 1F), hanged on a nylon rope each a plastic pin (Figure 1G,H), and used for the subsequent rearing study as group A (pro-male population) and group B (pro-female population). Both groups were separately hanged on ropes and re-reared for extra nine months until the next reproductive period (Figure 1I, Table 1). Sampling for sexing in the subsequent reproductive period was performed in October (16 months of age) and December 2016 (19 months of age). For all specimens sampled, shell length (SL) and softbody weight (BW) were measured. Then, the softbody and gonad were dissected, weighed for calculation of gonad index (GI; 100 × gonad weight/softbody weight (%)), and sampled for fixation for sexing by subsequent histological analysis. The sampled gonads were fixed with Davidson’s solution (artificial sea water/glycerin/formalin/ethanol/acetic acid, 3:1:2:3:1, v/v) at 4 °C for 24 h, rinsed with distilled water, dehydrated with an ascending ethanol series, and then embedded in paraffin. Cross sections, 6 μm-thick, were prepared, attached onto glass slides (Matsunami Glass, Tokyo, Japan), and used for hematoxylin and eosin (HE) (Muto Pure Chemicals, Tokyo, Japan) staining. Histological observation was performed for the specimens sampled in October and December in 2016. Because, in October, the gonad color was at the beginning of the reproductive period and still faint, we carefully evaluated the sex by histological observation.

### 2.2. Sample Preparation for mRNA Expression Analyses

One- to two-year-old farmed scallops (*M. yessoensis*) were purchased several times from local commercial suppliers (Mutsu Bay, Aomori, and Ogatsu Bay, Miyagi Prefecture, Japan) from September 2016 to March 2017. The gonads were sampled and stored in RNA*later* stabilization solution (Thermo Scientific, Waltham, MA, USA) at −30 °C for subsequent RNA extraction. At the same time, another piece of gonad was fixed in Davidson’s fixative overnight at 4 °C and processed as formalin-fixed paraffin-embedded tissue for in situ hybridization (ISH) detection with HE staining. Total RNA was extracted from various tissues using RNeasy mini kit (Qiagen, Tokyo, Japan) in accordance with the manufacturer’s protocol and quantified by spectrophotometry with a NanoDrop ND-1000 instrument (Thermo Scientific). RNA integrity was assessed by electrophoresis on a 1% (w/v) agarose gel. Total RNA (1 μg) was transcribed to cDNA using high-capacity cDNA reverse-transcription kits (Life Technologies, Tokyo, Japan).

### 2.3. Transcriptomic Survey and cDNA Cloning

A transcriptomic survey for *dmrt*s and *foxl2* was conducted by local blasting with the Yesso scallop transcriptome datasets (SRX047537 [24] and our previous data [25]). Known protein sequences reported in related species (e.g., Akoya pearl oyster, *C. farreri*, Pacific oyster, limpet) were used as queries, and candidate unigenes were carefully assessed by several bioinformatic analyses.

### 2.4. Bioinformatic Analyses

Deduced amino acid sequences of Dmrts and Foxl2 were generated from candidate contigs obtained from the above transcriptomic survey. Amino acid sequences were aligned by Clustal W2 and used for Bayesian inference (MrBayes v3.1.2, mrbayes.csit.fsu.edu) using a mixed model of amino acid substitutions (1,000,000 generations, sampling every 10th generation, and burn-in for the first 10,000 trees). Graphical representations of the phylogenic trees were obtained with FigTree (http://tree.bio.ed.ac.uk/software/figtree/). Domain structure analysis was also performed using SMART (http://smart.embl-heidelberg.de/).

### 2.5. Semi-Quantitative RT-PCR Assay

Total RNA and cDNA were prepared from the gonads during the early differentiating stage of the adult Yesso scallops in November 2015, as described previously [11]. Semi-quantitative RT-PCR was performed with GSP sets (Table 2) with Takara Ex Taq HS (TaKaRa-Bio, Kusatsu, Shiga Prefecture, Japan). Thermocycling parameters were 95 °C for 5 min, followed by 35 cycles (*dmrt*s: Unigene22131, Unigene26880, and Unigene30667) or 30 cycles (*my-foxl2*) of 30 s at 95 °C, 30 s at 58 °C, and 1 min at 72 °C, with a final elongation step of 72 °C for 5 min. The PCR products were electrophoresed on a 2% (w/v) agarose gel and photographed. The PCR amplicons were cloned into pGEM-T Easy vectors (Promega, Madison, WI, USA), and the cloned plasmids were extracted with Zyppy plasmid miniprep kit (Zymo Research, Irvine, CA, USA) and sequenced (Macrogen, Seoul, South Korea).

### 2.6. ISH Detection

Testis (GI = 10.6%) and ovary (GI = 9.7%) were excised from the adult Yesso scallops as for semi-quantitative PCR (SL of 112 ± 3 cm, softbody weight of 84 ± 6 g, GI of 9.3 ± 1%, mean ± SD, *n* = 9). The sliced gonads were fixed in Davidson’s solution at 4 °C for 24 h, rinsed with distilled water, dehydrated with an ascending ethanol series, and then embedded in paraffin. Cross sections were prepared at 6 μm thickness, attached onto FRONTIER glass slides (Matsunami Glass, Osaka, Japan), and used for ISH analysis, as described elsewhere [26]. Digoxigenin-labeled sense and anti-sense RNA probes for *my-dmrt2* and *my-foxl2* were individually transcribed in vitro using SP6 or T7 RNA polymerase (Roche, Mannheim, Germany) from the corresponding amplified DNA fragments (Table 2). The sections were permeabilized with proteinase K (5 μg/mL for 8 min at RT) (Roche), acetylated, and incubated with a hybridization mixture (0.5 μg/mL cRNA probe, 50% formamide, 2× saline sodium citrate (SSC) (pH 4.5), 50 μg/mL transfer RNA (tRNA), 50 μg/mL heparin, 0.02% sodium dodecyl sulfate (SDS), and 10% dextran sulfate). After hybridization at 70 °C for 16 h, the sections were washed in 0.2× SSC at 70 °C for 20 min in three times. Nonspecific binding probes were digested using RNase A solution (20 μg/mL) (Sigma-Aldrich Japan, Tokyo, Japan) and washed in 0.2× SSC at 70 °C for 20 min three times. The sections were blocked with Blocking One (Nacalai Tesque, Kyoto, Japan). Hybridized digoxigenin (DIG)-labeled probes were immunodetected with alkaline phosphatase-conjugated digoxigenin antibody (anti-DIG-AP Fab fragments, diluted 1:500). The AP-labeled sections were subjected to chromogenic reaction with nitro blue tetrazolium chloride (NBT)/5-bromo-4-chloro-3-indolyl phosphate, toluidine salt (BCIP) solution at RT in the dark. After color development, the sections were mounted in embedding medium (Softmount; Wako Pure Chemical Industries, Tokyo, Japan) and observed under a BX-53 microscope (Olympus, Tokyo, Japan).

### 2.7. qPCR Assay

Total RNA and cDNA were prepared from gonads during the maturation period of the adult Yesso scallops (*n* = 5 for each sex in September, October, and December 2016, and in January, February, and March 2017, farmed in Miyagi, Japan). The mRNA levels of *my-dmrt2*, *my-foxl2*, *my-soxb1*, *my-tesk*, and *my-vtg* were quantified in scallop testes and ovaries during the reproductive period using a qPCR system (7300 Real-time PCR system; Applied Biosystems, Warrington, UK), as described elsewhere [25], with specific primer sets (Table 2). The program was initially set at 50 °C for 2 min and 95 °C for 10 min, followed by 40 cycles of 15 s at 95 °C and 60 s at 60 °C. Dissociation curve analysis was set at 95 °C for 15 s, 60 °C for 60 s, and 95 °C for 15 s. The cycle threshold values were set at 0.2 to define the level of arbitrary fluorescence intensity on the 7300 System SDS software. Amplification efficiency (E, %) was calculated from a standard curve constructed from a diluted series of pooled cDNA samples (1:1, 1:2, 1:4, 1:8, and 1:16). Three stable reference genes (DEAD-box RNA helicase (*heli*), ubiquitin (*ubq*), and 60s ribosomal protein L 16 (*rpl16*)), studied by Feng et al. [27], were validated with all gonad cDNA specimens sampled. Using geNorm software (https://genorm.cmgg.be/), the two best reference genes (*rpl16* and *heli*) were selected among all three genes (geNorm stability values (M) were 0.617, 0.632, and 0.716 for *rpl16*, *heli*, and *ubq*, respectively) and used for normalization. The mRNA expression levels were calculated using the relative standard curve method with the normalization factors calculated above. For the comparison of means within the same sex, one-way ANOVA was used. If the one-way ANOVA was significant, Tukey’s multiple comparison test was used as a post-hoc test. For one-way ANOVA tests, significance levels were set at *p* < 0.05. For the comparison of means between sexes at a particular sampling point, two-way ANOVA was used. If the two-way ANOVA was significant, Bonferroni post-tests were performed.

## 3. Results

### 3.1. Tracking of the Sex Phenotype in the Yesso Scallops between One and Two Years of Age

**Figure 2:**
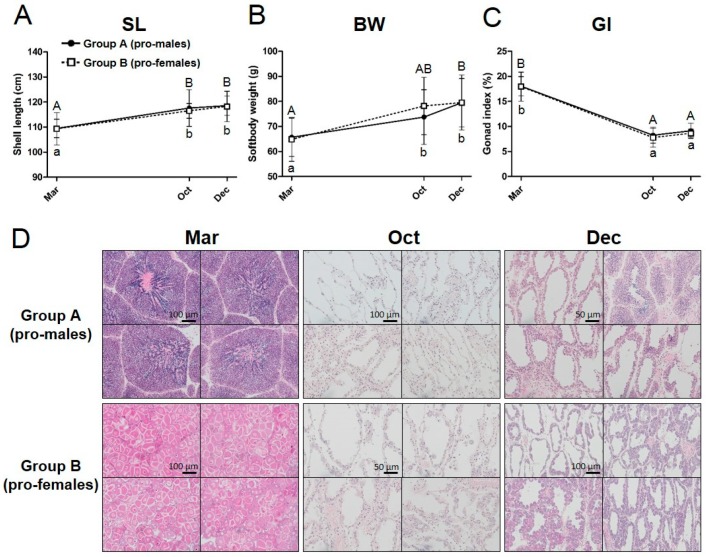
Growth and reproductive histories of the two-year-old Yesso scallops during the tracking experiment. Details of the body growth are shown for (**A**) shell length (SL), (**B**) softbody weight (BW), and (**C**) gonad index (GI). Lines with dots and dashed line with boxes show the data of group A (pro-males) and group B (pro-females) populations, respectively. Error bars show SEM at each sampling point (*n* = 10 for each group). Different superscript letters indicate significant differences within sex (lower case for pro-females and upper case for pro-males). (**D**) Representative histological observations for both groups are shown at each sampling point. Hematoxylin and eosin (HE) staining was performed for the gonads of pro-males and pro-females at different stages. March (Mar): Group A (pro-males) have mature testes. Testes are mainly filled with meiotic germ cells (e.g., spermatocytes and spermatids). Sperm are found in the center area of germinal acini. Group B (pro-females) have ovaries filled with full grown oocytes. October (Oct): Both group A (pro-males) and group B (pro-females) possess a small number of spermatogonia and oogonia, respectively. A few primary oocytes are observed in germinal acini of group B (pro-females) distinguishing it from group A (pro-males). December (Dec): Group A (pro-males) mainly possess proliferating spermatogonia, while group B (pro-females) possess growing oocytes.

To analyze the sex phenotype in the next reproductive period, we planned a tracking experiment (Figure 1A). We purchased and sexed the cultured Yesso scallops at 10 months of age in March 2016. During the sexing, we confirmed that most scallops possessed colored gonads, indicating that they reached maturity with sex-differentiated gonads (Figure 1B–D). In the beginning of the experiment (March 2016), the scallops were 10 months old, and the sex proportion of 300 scallops was 140:150 (males/females), showing an equal sex ratio (Table 1). Approximately, 10 scallops were not counted because of uncertain sexing. Next, we set up two rearing ropes for each sexed scallop population and performed additional rearing until the next reproductive season to confirm their subsequent sex phenotype (Figure 1I). During the following nine months of rearing, the scallops exhibited normal increases in shell size (Figure 2A) and softbody weight (Figure 2B). The GI dropped from the beginning of the fully mature phase (Figure 2C). No sex differences in growth and reproductive histories were observed (Figure 2A–C). At 16 months of age in October 2016 (six months later), the histological analysis was performed for each group (*n* = 10 for each group) and identified that all individuals of each group exhibited the initial sex as identified at 10 months of age (Table 1, Figure 2), at the beginning of the maturation stage (October in Figure 2D). At 19 months of age in December 2016 (nine months later), they reached the fully mature phase (Dec in Figure 2D). Another histological analysis identified that they all maintained their initial sex phenotype (*n* > 21 for each group) (Table 1).

### 3.2. Characterization of dmrts and foxl2 in the Yesso Scallops

**Figure 3:**
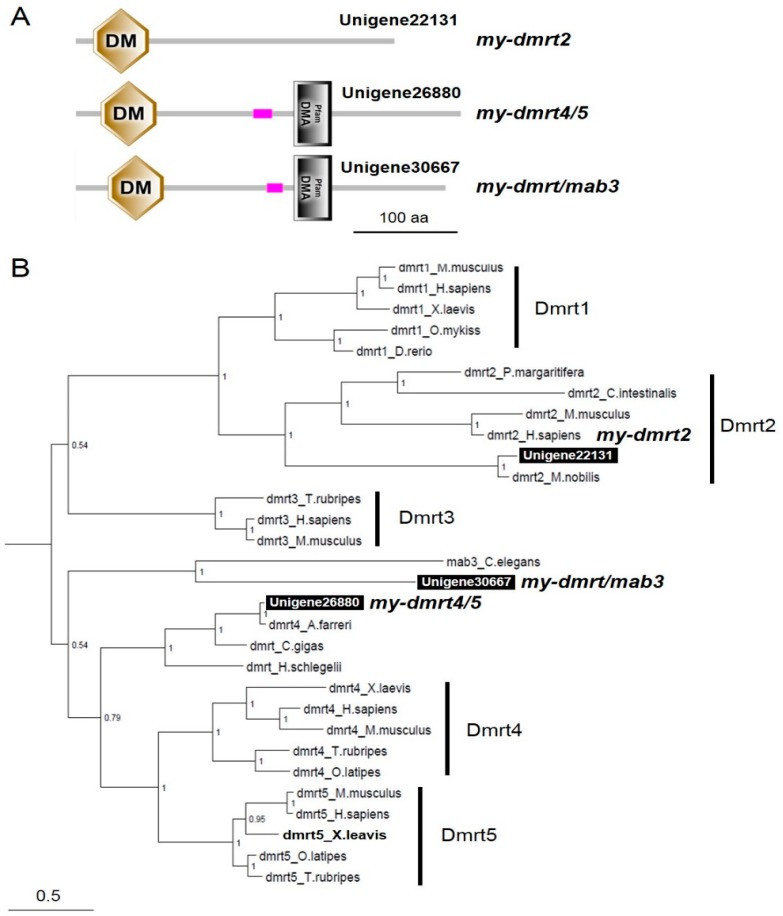
Domain analysis and phylogenic tree of *my-dmrt*s. (**A**) Domain structure analysis of *my-dmrt*s by SMART. The Doublesex/Mab-3 (DM) and DMRTA-specific C-terminal (DMA) domains are indicated. The scale bar represents a length of 100 amino acids. (**B**) The numbers at the nodes indicate Bayesian posterior probability. The scale bar indicates amino acid substitutions per site. GenBank accession numbers for the sequences are listed in Table S1.

**Figure 4:**
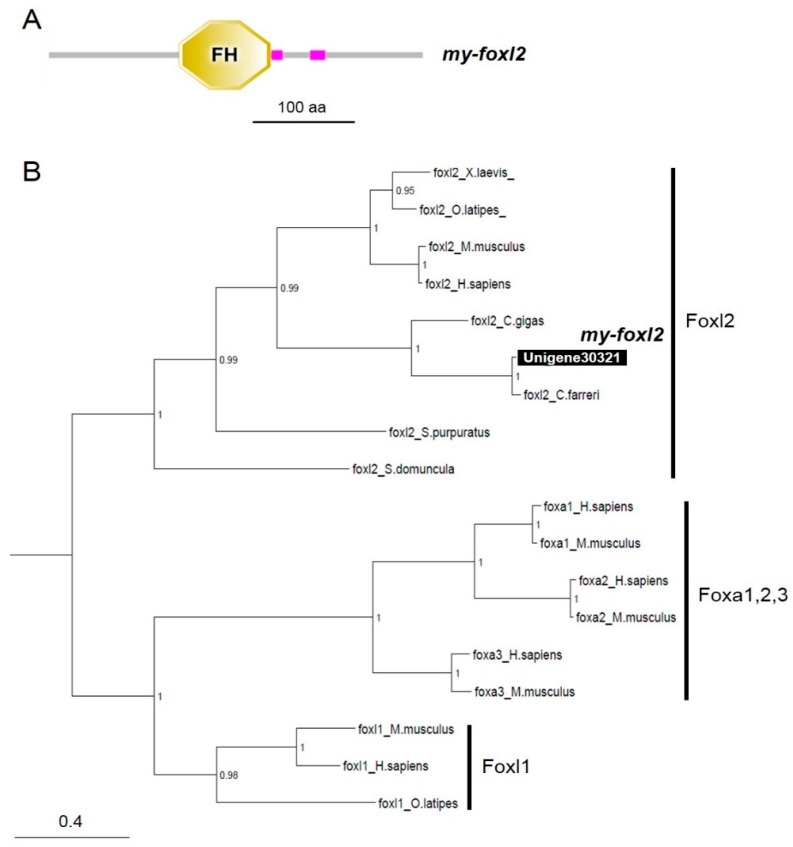
Domain analysis and phylogenic tree of *my-foxl2*. (**A**) Domain structure analysis of *my-foxl2* by SMART. The forkhead (FH) domain is indicated. The scale bar represents a length of 100 amino acids. (**B**) The numbers at the nodes indicate Bayesian posterior probability. The scale bar indicates amino acid substitutions per site. GenBank accession numbers for the sequences are listed in Table S2.

By performing local blasting with the Yesso scallop transcriptome datasets, we found three and one contigs of *dmrt* and *foxl2* cDNAs, respectively. Three *dmrt* and one *foxl2* candidate contigs that mostly covered open reading frames were obtained. Next, we confirmed their sequence by blasting with the genome sequence resource deposited in NCBI [28]. Sequences identical to the above contigs were also identified in NCBI resources (GenBank accession numbers are presented in Table 2; *my-dmrt2*_Unigene22131: XM_021498039, *my-dmrt4-5*_Unigene26880: XM_021521599, *my-dmrtmab3*_Unigene30667: XM_021513113, *my-foxl2*_Unigene30321: XM_021497746). Domain analysis revealed that three Dmrt candidates had a Doublesex/Mab-3 (DM) domain at the N terminal and two had a DMRTA-specific C-terminal (DMA) domain (Figure 3A). The Foxl2 candidate had a Forkhead (FH) domain, which acts as a sequence-specific DNA-binding transcription factor (Figure 4A).

Bayesian phylogenic reconstruction visualized the phylogeny of both Dmrts (Figure 3B) and Foxl2 (Figure 4B). The vertebrate Dmrts were generally separated into five clusters (Dmrt1 to 5). Notably, Unigene223131 (*my-dmrt2*) and *Mimachlamys nobilis Dmrt2* were positioned in the subclade of Dmrt2. However, regarding the details for other invertebrate Dmrts, two clusters were generated. One cluster was formed with bivalve *Dmrts* containing Unigene26880 (*my-dmrt4/5*), with the closest relationship to *Chlamys (Azumapecten) farreri* Dmrt4. This cluster was closer to vertebrate *Dmrt4*/5. Another cluster was formed with *Caenorhabditis elegans* Mab3 with Unigene30667 (*my-dmrt/mab3*) showing a distant relationship with vertebrate Dmrts. For Foxes, three clusters (Foxl1, Foxl2, and Foxa1-3) were generated (Figure 4B). Unigene30321 (*my-foxl2*) was subclustered in bivalve Foxl2s within the Foxl2 cluster including vertebrates.

### 3.3. Validation of Molecular Markers of Sex Identification for Testis/Male or Ovary/Female in the Adult Yesso Scallop

**Figure 5:**
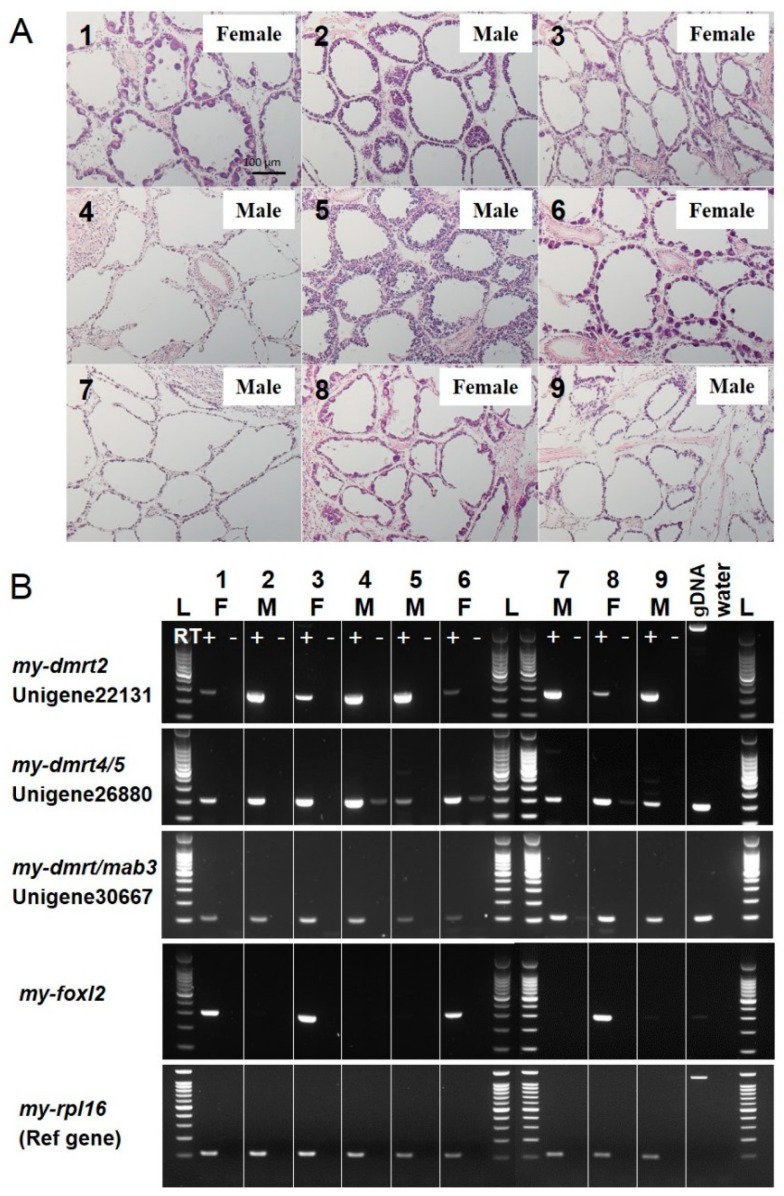
Distribution of *my-dmrt2* and *my-foxl2* mRNAs between ovaries and testes in the Yesso scallop at the early differentiating stage. Nine specimens were subjected to histological analysis (**A**) and semi-quantitative RT-PCR (**B**). (**A**) HE staining was performed for the above assessed gonad specimens. The numbers are as for the specimens analyzed in RT-PCR. At this stage, males and females possess proliferating spermatogonia and immature oocytes along the wall of germinal acini. Females are distinguished from males by the presence of oocytes. (**B**) Gonad cDNAs and RT controls (without reverse transcriptase) for females (*n* = 4) and males (*n* = 5) were analyzed. Genomic DNA and water samples were used as negative controls; *my-rpl16* was used as an endogenous reference gene. Amplicon sizes in base pairs are indicated in Table 2.

Sex-specific mRNA expression of *my-dmrt*s and *my-foxl2* in the gonads was subjected to RT-PCR analysis with testis and ovary cDNAs of adult scallops. To evaluate sex identification markers, we chose gonads at the early differentiating stage as specimens, because their sex could not be distinguished by visual judgment of gonad color (data not shown). For the nine Yesso scallops, histological observation of the gonads was first performed to carefully distinguish their sex (Figure 5A). Then, RT-PCR screening for *my-dmrts* and *my-foxl2* candidates was performed (Figure 5B). For three *my-dmrt*s, Unigene22131 (*my-dmrt2*) showed dominant expression in the testes rather than in the ovaries. Two other *dmrt*s (i.e., Unigene26880 (*my-dmrt4/5*) and Unigene30667 (*my-dmrt/mab3*)) showed uniform expression in testes and ovaries. In addition, Unigene30321 (*my-foxl2*) was specifically expressed in the ovaries, showing no mRNA expression in the testes.

**Figure 6:**
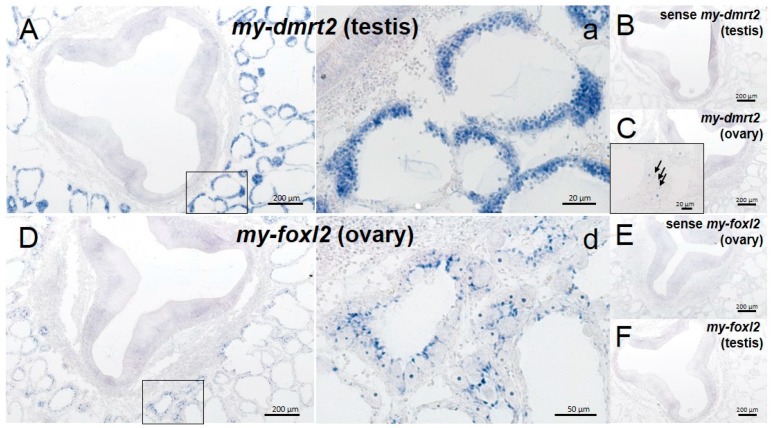
Localization of *my-dmrt2* and *my-foxl2* mRNAs in the Yesso scallop gonads. In situ hybridization (ISH) staining for *my-dmrt2* (anti-sense probe, **A** and **C**; sense probe, **B**) and *my-foxl2* (anti-sense probe, **D** and **F**; sense probe, **E)** for testis and ovary. Black boxes in (**A**) and (**D**) indicate areas where high-magnification images were captured (**a** and **d**). Inset in C is a high-magnification view showing the faint signals of *my-dmrt2* in ovarian cells (arrows).

ISH detection supported the sex-specific expression of *my-dmrt2* and *my-foxl2* for testis and ovary, respectively. At the early differentiating stage, the testes were filled with proliferating spermatogonia, whereas the ovaries were filled with growing primary oocytes and fewer oogonia (Figure 6). As a testis/male-specific marker, *my-dmrt2* mRNA was detected in spermatogonia in the testis (Figure 6A-a), while its faint signals were seen in some ovarian cells (inset in Figure 6C). Regarding ovary/female-specific markers, *my-foxl2* mRNA was detected in follicle cells attached to growing oocytes in the ovary, and its expression was absent in testicular cells (Figure 6D-d,F). In addition, no signal was observed in sense probe conditions for *my-dmrt2* and *my-foxl2* in both testis and ovary (Figure 6B,E).

### 3.4. Expression Profiles of Sex Identification Markers in the Yesso Scallop Gonads during the Reproductive Cycle

**Figure 7:**
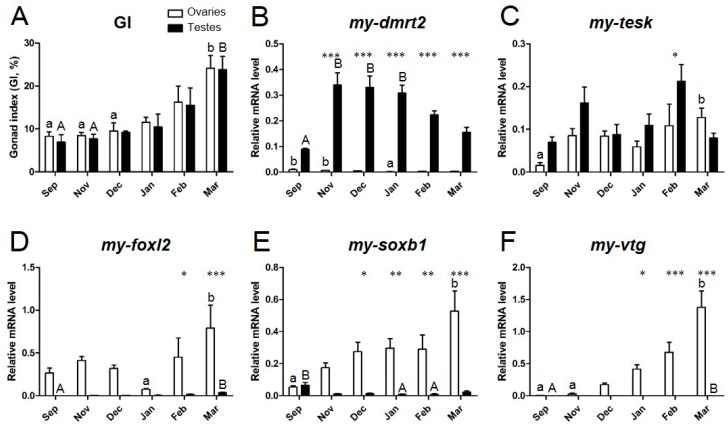
History of (**A**) GI and quantification of (**B**) *dmrt2*, (**C**) *tesk*, (**D**) *foxl2*, (**E**) *soxb1*, and (**F**) *vtg* mRNAs in the gonads of the Yesso scallops throughout the reproductive cycle. White and black bars show the relative mRNA levels in testes and ovaries, respectively. Error bars show SEM at each sampling point (*n* = 5 for each sex). Different superscript letters indicate significant differences within sex (lower case for ovaries and upper case for testes). Asterisks *, **, and *** indicate significant differences between testes and ovaries at *p* < 0.05, *p* < 0.01, and *p* < 0.001, respectively.

During the reproductive cycle, the mRNA expression of *my-dmrt2*, *my-foxl2*, *my-soxb1*, *my-tesk*, and *my-vtg* in testes and ovaries was quantified by real-time qPCR (Figure 7). The maturation stages were defined by GI (GI in Figure 7A) as follows: September (early sex differentiation), November (growing), December (early mature), January (middle mature), February (fully mature), and March (fully mature and spawning), as reported elsewhere [11]. *my-dmrt2* was consistently expressed at a higher level in testes than in ovaries throughout the reproductive cycle (Figure 7B). The mRNA expression of *my-dmrt2* in testes drastically increased in November after gonad sex differentiation in September. The mRNA expression of *my-dmrt2* gradually decreased until the fully mature stage in March. The mRNA expression of *my-tesk* showed no sex-biased pattern throughout the reproductive cycle (Figure 7C). In detail, *my-tesk* mRNA was expressed at a higher level in testes in November and February, whereas no different levels of mRNA were observed in ovaries. In contrast, *my-foxl2* mRNA was expressed at a higher level in ovaries for all maturational stages, while a much lower expression was seen in testes (Figure 7D). Fully mature ovaries in March showed the highest mRNA expression of *my-foxl2* throughout the reproductive cycle. The mRNA expression of *my-soxb1* was ovary-dominant during maturation (Figure 7E), similar to the mRNA expression of *my-foxl2*, gradually increased until the fully mature stage in March, according to gonad maturation, and showed the highest level in fully mature ovaries in March. *my-vtg* mRNA was dominantly expressed in ovaries, where its expression level gradually increased according to gonad maturation, peaking in fully mature ovaries in March (Figure 7F). No expression of *my-vtg* mRNA was observed in testes throughout the reproductive cycle.

## 4. Discussion

### 4.1. Phenotypic Stability of Sex in the Yesso Scallop

The present study is the first to report on the analysis of phenotypic stability of sex after the sex differentiation phase in the Yesso scallop. In most situations, one-year-old Yesso scallops exhibited a 1:1 sex ratio under culture conditions [2,4,5]. Kawamata [5] reported the sex differentiation pattern in the Yesso scallop. After birth, all scallops differentiate into males, with spermiation at 4–5 months of age. At 7 months, some of the males enter a regressing phase of testis where sperm are phagocytized and then start a transition process with the generation of ovarian germ cells in germinal acini. At 8 months of age, approximately 35% and 57% of scallops are males and females, respectively. The remaining 8% are hermaphrodites. It was proposed that hermaphrodites found before one year of age are in a transition phase of sex reversal. In addition, it was suggested that females do not directly differentiate from sexually immature scallops. However, no study has traced the history of sex reversal in the same individual over the first three years after birth to confirm the following points: (i) How does the hermaphroditic gonad arise from the testis? (ii) Does the hermaphroditic gonad differentiate into a normal ovary? To resolve these issues, tag tracking to confirm the sexual fate in the subsequent reproductive season is an optimal approach.

**Figure 8:**
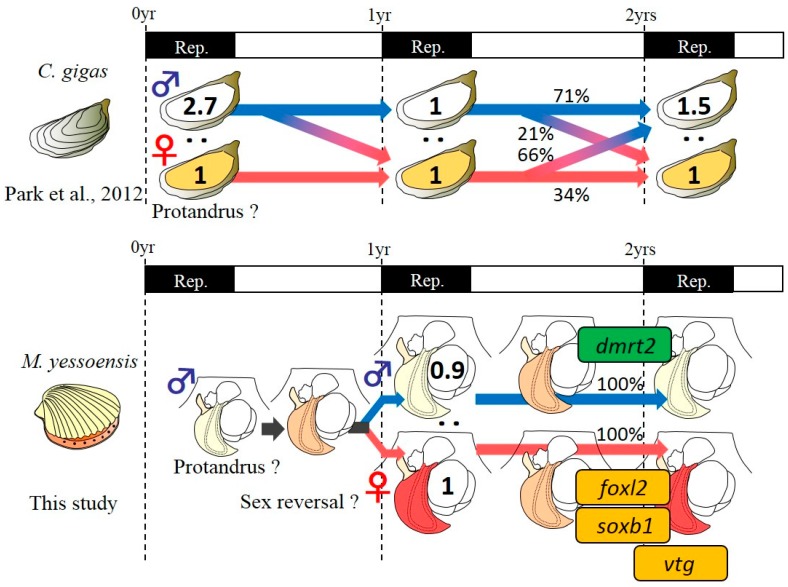
Scheme of phenotypic plasticity of sex in two bivalve species from tracking studies and expression of sex identification markers. Pacific oyster *Crassostrea gigas* exhibits an alternative sexuality [7] while the Yesso scallop *Mizuhopecten yessoensis* shows a sex-stable sexual maturation from one to two years of age. In the scallop, for male differentiation, *my-dmrt2* is dominantly expressed in the male gonads. In contrast, for female differentiation, *my-foxl2*, *my-soxb1*, and *my-vtg* are consistently expressed at a higher level in the female gonads.

In the present study, we conducted confirmatory sexing with sexually mature scallops cultured in Ogatsu Bay (Miyagi Prefecture, Japan) at one year of age. The experiment with accurate judgments of sex identified an almost 1:1 sex ratio (females/males, 1:0.93) in 300 individuals. This equal sex ratio indicates the completion of the first sex differentiation at one year of age, as similarly observed in several studies [2,4,5]. In the present study, additional rearing (nine months) was performed for both female and male populations to confirm their subsequent sex phenotype in the next reproductive season at two years of age. Surprisingly, after the additional nine months of rearing, both female and male populations at two years of age exhibited the same sex phenotype as determined at one year of age. This is the first observation that the Yesso scallops maintained their sex phenotype after their first sex differentiation, indicating that their sex does not fluctuate depending on external factors, unlike that of the Pacific oyster [7] (Figure 8).

In Pacific oyster, the morphological sex of the gonad is affected not only by genetics but also by environmental factors [29]. Oysters cultured under food-rich conditions often show female-biased sex differentiation, in contrast to wild oysters under an oligotrophic environment [30]. Therefore, a second sex reversal was observed in Pacific oyster, namely, from male to female from one to two years of age, then from female to male from two to three years of age [7]. It was proposed that the Pacific oyster exhibits flexibility in terms of sex differentiation. In contrast, in the present study, it is proposed that the Yesso scallop has very low sex plasticity after sex differentiation. The sex ratio in this scallop may thus be less influenced by food availability; food-rich conditions may promote differentiation to females. However, no study has shown that the proportion of females was more than double that of males in any rearing condition. These observations indicate that the sex of this scallop is not notably influenced by food availability, unlike that of oysters, suggesting that sex determination in the Yesso scallop is under strict genetic control.

In addition, Kawamata [5] reported that the timing of sex differentiation among different aquaculture sites (i.e., Lake Saroma, Funka Bay, and Mutsu Bay) was slightly different, particularly the timing of the appearance of females. Specifically, at Lake Saroma, the timing of sex differentiation was brought forward for a decade starting from 1978. We believe that this shift to early maturation was caused by eutrophication, resulting in an increase in phytoplankton. If so, food-rich conditions accelerate sex differentiation for both females and males. In addition, Wakui and Obara [2] reported that one-year-old scallops in Lake Saroma were all male, suggesting that food-scarce conditions result in an abundance of males. However, taken together, these observations do not indicate that food-rich conditions increase the ratio of females to males but, rather, that they simply accelerate ovarian gonad differentiation. Hence, females were observed earlier, but the sex ratio was always equal under different culture conditions at most aquaculture sites. These findings suggest that male sex differentiation requires less energy than female sex differentiation, as observed for teleosts, suggesting that oogenesis also involves higher energy consumption and longer maturation time in the Yesso scallop.

### 4.2. my-dmrt2 as a Testis Marker in the Yesso Scallop

To describe the process of gonad sex differentiation in the Yesso scallop, molecular markers are crucial for understanding the molecular basis of gonad development, as proposed [23]. In the present study, we aimed to characterize *dmrt* and *foxl2* as markers of testis or ovary differentiation, respectively. To identify all paralogs and/or isoforms of both *dmrt* and *foxl2*, we performed a transcriptomic survey using a local blast system, as reported elsewhere [25]. Our survey identified three paralogs of *dmrt* and one of *foxl2* from the Yesso scallop transcriptome datasets.

For invertebrate *dmrt*s, Chen et al. [31] summarized the genetic diversity of *Dmrt* members in metazoans and reported that mollusks should have four *dmrt* paralogs. Interestingly, Bellefroid et al. [32] reported that the snail *Lottia gigantea* possesses four Dmrt paralogs with a DM domain, and three out of four Dmrt paralogs have a DMA domain, called the DMRTA (DMRTA-specific C-terminal) motif [33]. For *my-dmrt*s found in the Yesso scallop, all three *my-dmrt* paralogs possessed a DM domain, but *my-dmrt2* (Unigene22131) only lacked a DMA domain, as found in vertebrate *Dmrt1* and *Dmrt2* [32], although two other scallop *my-dmrt* paralogs possessed a DMA domain, which is a typical domain of DMRTA proteins whose function is unknown (Pfam; http://pfam.xfam.org). Roles of DMRTA proteins (e.g., Dmrt3, Dmrt4, and Dmrt5) in neurogenesis and patterning of the developing nervous system have been proposed in vertebrates [32]. Notably, our RT-PCR analysis identified that *my-dmrt2* showed testis-dominant expression, whereas two other *my-dmrt*s exhibited no sex-biased mRNA expression. Among three *my-dmrt*s, none showed ovary-dominant mRNA expression, unlike in stony coral [31]. *Dmrt1* is well known to be involved in male sex determination and differentiation in a wide range of vertebrates [34,35]. In contrast, this study reports that *my-dmrt2*, which was named *DMRT1L* [23], showed a testis-dominant expression pattern similar to those of vertebrate *dmrt1* and Akoya pearl oyster *dmrt2* [36]. Because no *dmrt1* has been found in mollusks [17,31], we believe that *my-dmrt2* is a functional ortholog of vertebrate *dmrt1*. Our ISH analysis revealed that *my-dmrt2* mRNA was localized in male germ cells (i.e., spermatogonia), differently from a previous report [23]. Similar germ cell-dominant expression was seen in Akoya pearl oyster [36] and for zebrafish *dmrt1* [37], *dmrt3* [38], and *dmrt5* [39], whereas Sertoli cell-specific expression of *dmrt1* was observed in teleosts [40,41] and a tetrapod [42]. In addition, human Dmrt1 showed dynamic expression in both Sertoli cells and spermatogonia [34]. These observations suggest the genetic divergence of *dmrt*-led molecular regulation for sex determination and differentiation in a species-specific manner.

Our qPCR results indicate that *my-dmrt2* was dominantly expressed in testes throughout the reproductive period and showed a higher expression level in November (growing) and December (early mature) when spermatogonia were mainly proliferating [11]. However, *my-tesk* did not show consistent sex-biased expression [43]. Therefore, *my-dmrt2* is a marker of testis differentiation in the Yesso scallop. Because *my-dmrt2* was detected in the spermatogonia from the early differentiating stage, this marker is applicable for sexing at an early stage of sex differentiation.

### 4.3. my-foxl2 as an Ovary Marker in the Yesso Scallop

For *my-foxl2*, our in silico survey found one candidate contig with a high e-value, indicating that the Yesso scallop has no other *foxl2* sub-isoforms. Our RT-PCR analysis indicated that *my-foxl2* showed ovary-specific expression, with no expression detected in testes. In contrast, *my-dmrt2*, a testis marker, was slightly expressed in ovaries. This slight expression may occur in oogonia, but our ISH detection method did not identify it, being it under the detection limit. Notably, ISH detection revealed that *my-foxl2* mRNA was specifically localized in the ovarian follicle cells attached to oocytes, and no expression was seen in germ cells, differently from what previously reported [23]. To the best of our knowledge, this is the first time that a strict control of the expression of *my-foxl2* in ovarian follicle cells is observed in bivalve species, and it implies appropriate regulation of sex differentiation in the Yesso scallop. For instance, Pacific oyster *foxl2* (*cg-foxl2*) mRNA was detected in male and female gonads [44]. Specifically, *cg-foxl2* mRNA was localized in spermatogonia to spermatids in male gonads and in oogonia to vitellogenic oocytes in female gonads. In addition, Zhikong scallop (*C. farreri*) *foxl2* (*cf-foxl2*) mRNA was seen not only in follicle cells but also in ovarian and testicular germ cells [21]. By comparison of the above bivalve *foxl2* mRNA localizations, it appears that the Yesso scallop has a robust system of female differentiation led by *foxl2* expression. In addition, although previous studies [21,44] have suggested the possible presence of natural antisense mRNA of *foxl2* in bivalve gonadal cells, the present study identified no such signal.

Our qPCR results revealed that *my-foxl2*, *my-soxb1*, and *my-vtg* were consistently expressed at a higher level in ovaries throughout the reproductive period, and their mRNA levels generally increased according to ovarian maturation, indicating their potential for use as ovary markers. Among the above three genes, *my-foxl2* is a more appropriate marker for the early differentiating stage for sexing. In contrast, *my-vtg* would be a useful indicator of oocyte maturation stage during the late stage of maturation [45]. Surprisingly, *my-soxb1* showed ovary-dominant expression in the present study. Because Sox transcriptional factors were characterized as Sry-related high-mobility group box members and found to be essential for testis development [46], *my-soxb1* was originally designed as a testis marker in a previous study, like *sox2* [43]. From the qPCR results, we believe that *my-soxb1* is a functional ortholog of *sox3* [47], which shows ovary-specific expression, as reported in Nile tilapia [48].

## 5. Conclusions

The present study focused on the phenotypic stability of sex in the adult Yesso scallop after sex differentiation, suggesting that this scallop is a sex-stable bivalve after sex differentiation and maintains a sex-stable maturation system throughout its life. Importantly, the present study assessed all *dmrt* paralogs identified from the transcriptome of the Yesso scallop and eventually characterized *my-dmrt2* and *my-foxl2* as testis and ovary markers, respectively. Notably, our ISH results provided for the first time sex-specific spatial expression patterns of *my-dmrt2* and *my-foxl2*, which have not been reported previously [23]. It is noteworthy that our qPCR validations were performed with the specimens that had a background regarding their sex phenotype in the last reproductive season. This advantage enabled the robust quantitative analysis especially for the early differentiating stage by avoiding incorrect judgements of sex using histological observation. Therefore, the qPCR-based sexing with sex identification markers reported in this study would be applicable to distinguish scallop sex throughout the reproductive cycle using biopsies of the gonads. We believe that the knowledge of molecular markers can be a powerful tool not only for the early evaluation of bivalve sex in broodstock management for seed production in shellfish aquaculture but also in fundamental research of bivalve gonad development and sex differentiation based on the control of neuroendocrinological regulation by sex steroids and neuropeptides [13,25,49,50,51].

## Figures and Tables

**Table 1:** Changes of sex ratio from one to two years of age in the Yesso scallop.

Sampling Period (Age)	Mar 2016 (10 Months)	Oct 2016 (16 Months)			Dec 2016 (19 Months)		
Group	Intact	Survivors	Analyzed	Female:Male	Survivors	Analyzed	Female:Male
Group A (pro-males)	140	45	10	0:10	22	22	22:0
Group B (pro-females)	150	51	10	10:0	21	21	0:21

**Table 2:** Gene names, type of PCRs, primer sequences, amplicon sizes (bp), intron insertion in the amplicon, GenBank accession numbers, r squared value (r^2^), PCR efficiency (E, %) and references for *my-dmrt2*, *my-dmrt4/5*, *my-dmrt/mab3*, *my-foxl2*, *my-sox*, *my-tesk*, *my-vtg*, *my-heli*, *my-rpl16*, and *my-ubq* in the Yesso scallop.

Gene	PCR Type	Sequence (5′–3′)	Amplicon (bp)	Intron Insertion	Genbank Acc. No.	r^2^	E (%)	Reference
*my-dmrt2* (Unigene22131)	RT-PCR, qPCR	Fw: TGAGCAATAACAAGGAGCTGTTAG	264	yes	XM_021498039	0.99	78.18	This study
		Rv: CTCAGGGCGATCTGTTCCTTTGA						
	ISH	Fw: TGAGCAATAACAAGGAGCTGTTAG	1026	-				
		Rv: TGTCATTCCTCAAGTAACCTTCAT						
*my-dmrt4/5* (Unigene26880)	RT-PCR	Fw: TGCCTACAGTGGACTCACGACGGAC	201	no	XM_021521599			This study
		Rv: TCTCAGGCTCCGTCCTTACAGGCG						
*my-dmrt/mab3* (Unigene30667)	RT-PCR	Fw: CTCAGAAGTCTGCAGCAACACAC	174	no	XM_021513113			This study
		Rv: CTCATGTTCGTACATTGCAAGCT						
*my-foxl2* (Unigene30321)	RT-PCR, qPCR	Fw: TCAGAGTCACTCGATAACCTTACT	304	no	XM_021497746	0.97	90.84	This study
		Rv: CCTGGCTGCTACACCGTACGCCACT						
	ISH	Fw: TCAGAGTCACTCGATAACCTTACT	828	-				
		Rv: TAGGGACCGCAGTGGTTGTCAGCA						
*my-soxb1*	qPCR	Fw: ACAAACTCTCGCAGGGGTAG	309	NT	KY523527	0.99	84.4	Otani et al., 2017
		Rv: GCACCGAGTCGCTTACTGAT						Yu et al., 2017
*my-tesk*	qPCR	Fw: CACGCCAAGGAATCTGAGG	472	NT	NA	0.99	111.14	Otani et al., 2017
		Rv: AGGATGTGAAGGGTCGTTG						
*my-vtg*	qPCR	Fw: CCTCTATGCCGGACATTTGC	100	NT	AB055960	0.97	83.03	Osada et al., 2004
		Rv: CAAAGCCACGCTGCTATCTTT						
*my-heli*	qPCR	Fw: CCAGGAGCAGAGGGAGTTCG	186	yes	NA	0.99	77.02	Feng et al., 2013
		Rv: GTCTTACCAGCCCGTCCAGTTC						
*my-rpl16*	RT-PCR, qPCR	Fw: CTGCCAGACAGACTGAATGATGCC	117	yes	NA	0.99	95.49	Feng et al., 2013
		Rv: ACGCTCGTCACTGACTTGATAAACCT						
*my-ubq*	qPCR	Fw: TCGCTGTAGTCTCCAGGATTGC	184	yes	NA	0.99	80.05	Feng et al., 2013
		Rv: TCGCCACATACCCTCCCAC						

## Supplementary Materials

The following are available online at https://www.mdpi.com/2076-2615/9/5/277/s1, Table S1: Genbank accession numbers of *dmrt* genes used in the phylogenic analysis, Table S2: Genbank accession numbers of *fox* genes used in the phylogenic analysis.
