# Erratum: Nagasawa, K. et al. Phenotypic Stability of Sex and Expression of Sex Identification Markers in the Adult Yesso Scallop *Mizuhopecten yessoensis* throughout the Reproductive Cycle. *Animals* 2019, *9*(5), 277

**DOI:** 10.3390/ani9090631

**Published:** 2019-08-29

**Authors:** Kazue Nagasawa, Tongchai Thitiphuree, Makoto Osada

**Affiliations:** Laboratory of Aquacultural Biology, Graduate School of Agricultural Science, Tohoku University, 468-1 Aoba, Aramaki, Aoba-ku, Sendai, Miyagi 980-8572, Japan

In the published article, “Phenotypic Stability of Sex and Expression of Sex Identification Markers in the Adult Yesso Scallop *Mizuhopecten yessoensis* throughout the Reproductive Cycle. *Animals* **2019**, *9*(5), 277. doi:10.3390/ani9050277” [1], the proportion numbers in Table 1 are inverted.

In Table 1, the proportion numbers “22:0” and “0:21” should be replaced with “0:22” and “21:0” (Table 1).

**Table 1:** Changes of sex ratio from one to two years of age in the Yesso scallop.

Sampling Period (Age)	Mar 2016 (10 Months)	Oct 2016 (16 Months)			Dec 2016 (19 Months)		
**Group**	**Intact**	**Survivors**	**Analyzed**	**Female:Male**	**Survivors**	**Analyzed**	**Female:Male**
Group A (pro-males)	140	45	10	0:10	22	22	0:22
Group B (pro-females)	150	51	10	10:0	21	21	21:0

The authors would like to apologize for any inconvenience caused to the readers by this change. The manuscript will be updated and the original copy will remain online on the article webpage.
